# Salivary cytokine profile in patients with ischemic stroke

**DOI:** 10.1038/s41598-021-96739-0

**Published:** 2021-08-25

**Authors:** Mateusz Maciejczyk, Kacper Maksymilian Mil, Piotr Gerreth, Katarzyna Hojan, Anna Zalewska, Karolina Gerreth

**Affiliations:** 1 Department of Hygiene, Epidemiology and Ergonomics, Medical University of Bialystok, 2C Adama Mickiewicza Street, 15‐022 Bialystok, Poland; 2 Students Scientific Club “Biochemistry of Civilization Diseases” at the Department of Hygiene, Epidemiology and Ergonomics, Medical University of Bialystok, 2c Mickiewicza Street, 15-233 Bialystok, Poland; 3 Private Dental Practice, 57 Kasztelanska Street, 60‐316 Poznan, Poland; 4 Postgraduate Studies in Scientific Research Methodology, Poznan University of Medical Sciences, 10 Fredry Street, 60-701 Poznan, Poland; 5 Department of Occupational Therapy, Poznan University of Medical Sciences, Swiecickiego Street 6, 60-781 Poznan, Poland; 6 Department of Rehabilitation, Greater Poland Cancer Centre, 15 Garbary Street, 61‐866 Poznan, Poland; 7 Experimental Dentistry Laboratory, Medical University of Bialystok, 24A Marii Sklodowskiej‐Curie Street, 15‐276 Bialystok, Poland; 8 Department of Risk Group Dentistry, Chair of Pediatric Dentistry, Poznan University of Medical Sciences, 70 Bukowska Street, 60‐812 Poznan, Poland

**Keywords:** Diagnostic markers, Predictive markers, Prognostic markers, Stroke

## Abstract

Inflammation plays a crucial role in stroke pathogenesis. Thus, it is not surprising that cytokines, chemokines, and growth factors have been advocated in stroke diagnostics. Our study is the first to evaluate the salivary cytokine profile in patients with ischemic stroke. Twenty-five patients with subacute ischemic stroke and an age-, sex-, and oral hygiene status-matched control group were enrolled in the study. The number of patients was set a priori based on our previous experiment (α = 0.05, test power = 0.9). Salivary concentrations of tumor necrosis factor α (TNF-α), interleukin 6 (IL-6), and interleukin 10 (IL-10) were assessed using an ELISA method. We showed that salivary TNF-α and IL-6 were significantly higher, whereas IL-10 content was statistically lower in both non-stimulated (NWS) and stimulated (SWS) whole saliva of ischemic stroke patients. However, evaluation of cytokines in NWS rather than in SWS may be of greater diagnostic value. Of particular note is salivary TNF-α, which may indicate cognitive/physical impairment in post-stroke individuals. This parameter distinguishes stroke patients from healthy controls and correlates with cognitive decline and severity of functional impairment. It also differentiates (with high sensitivity and specificity) stroke patients with normal cognition from mild to moderate cognitive impairment. Saliva may be an alternative to blood for assessing cytokines in stroke patients, although further studies on a larger patient population are needed.

## Introduction

Stroke is a clinical syndrome characterized by the sudden onset of focal and sometimes generalized brain damage with symptoms lasting longer than 24 h^1^. According to the World Health Organization (WHO), stroke is the third leading cause of death in the adult population^2^. It is estimated that 5.5 million people die annually from this disease, accounting for 10% of all deaths^3^. Stroke is also the leading cause of long-term functional impairment and disability in adults^4^. There are two types of stroke: 10–15% are hemorrhagic strokes, and the rest are ischemic strokes^5^.

Inflammation plays a crucial role in stroke pathogenesis^2,6,7^. Acute cerebral ischemia induces cellular-molecular mechanisms involving both the central nervous system (CNS) and the vascular system. In response to ischemic brain injury, acute inflammatory reactions are induced, mediated by various cytokines, chemokines, and growth factors^8^. Among them, a unique role is attributed to TNF-α (tumor necrosis factor α), which is the first mediator synthesized in response to acute cerebral ischemia^6^. During the acute phase of ischemic stroke, TNF-α is produced by leukocytes circulating in the peripheral blood and its expression is highest between 8 and 24 h after the onset of cerebral ischemia. Under these conditions, other pro-inflammatory cytokines are activated (e.g., interleukin-6 (IL-6) and interleukin-8 (IL-8)). TNF-α also upregulates the expression of matrix metalloproteinases (MMPs), which increases endothelial cell permeability and results in blood–brain barrier (BBB) dysfunction^9,10^. Pro-inflammatory mediators exert direct damaging effects on neurons and astrocytes and promote prostaglandin release and leukocyte migration^2,6,7^. It has been shown that IL-6 and TNF-α stimulate the synthesis and secretion of several chemotactic factors, which activate and attract peripheral blood leukocytes (neutrophils and monocytes) to the site of stroke lesions^9,11^. Therefore, adhesion molecules' expression on the endothelial surface is increased, which potentiates leukocyte penetration into the ischemic brain area. Simultaneously, the secretion of interleukin-10 (IL-10) increases, inhibiting the inflammatory response^12^.

Stroke diagnosis is based on physical examination and imaging studies (CT)^13^. Despite the precise definition of stroke, up to 20–25% of diagnoses may be conditions that mimic stroke^14^. Availability of the latest diagnostic techniques (CT perfusion, MRI) is also often limited^15^. Therefore, it is not surprising that new diagnostic methods are still being sought^16^. In particular, biochemical biomarkers are being searched extensively to facilitate early diagnosis of stroke or to establish further patient’s prognosis^17,18^. Given the critical role of inflammation in stroke pathogenesis, the use of cytokines and chemokines in laboratory diagnostics has been increasingly advocated^16,19^. Of particular note is the evaluation of TNF-α and IL-6, whose serum levels increase in the acute phase of stroke as well as correlate with its cerebrospinal fluid (CSF) levels and stroke focus size^20–23^. In postmortem studies, TNF-α positive cells are observed in all brains of patients with severe ischemic stroke 3 days after stroke and are present up to 15 months after indecent^24,25^. Similar relationships were observed for IL-6^24,25^. Interestingly, using multiple logistic regression, plasma IL-6 was shown to be an independent factor for early clinical deterioration in stroke patients. This association was statistically significant in all subtypes of ischemic stroke, as well as in patients with cortical and subcortical infarction^26^. The high diagnostic utility also seems to be demonstrated by IL-10, whose levels decrease in patients with ischemic stroke and indicate the predominance of pro-inflammatory processes in the early stroke phase^12,27^.

Nowadays, the use of biological materials collected in a non-invasive manner, without special equipment and medical personnel, is gaining interest in laboratory diagnostics^28,29^. This reduces the patient's anxiety associated with the examination, promotes more frequent monitoring of health condition, and thus diagnoses the disorder at an early stage^30^. Saliva is an interesting alternative to blood or CSF that are commonly used in laboratory medicine^28,31^. Saliva is a secretion of major and minor salivary glands containing water (94–99%) and numerous proteins, lipids, carbohydrates, hormones, immunoglobulins, cytokines, growth factors, and vitamins^32–34^. Its composition is therefore very similar to blood plasma. Indeed, the level of many compounds in saliva correlates with their content in blood/CSF, indicating a solid diagnostic potential^28,31,35–37^. Although salivary cytokines are used to diagnose many systemic diseases^38–42^, there is no data on salivary inflammatory mediators' diagnostic utility in stroke individuals. Considering the continuous increase in stroke morbidity/mortality and the lack of non-invasive biomarkers of the disease, our study aimed to evaluate TNF-α, IL-6, and IL-10 levels in the non-stimulated (NWS) and stimulated (SWS) whole saliva of stroke patients.

## Material and methods

### Study population

The study was carried out from June to August 2019 in a health center (Bonifraterskie Centrum Zdrowia) in Piaski–Marysin (Piaski, Poland). In this center, patients with various disorders are hospitalized, including the individuals after the cerebral stroke that arrive from different country provinces. At the time of the research, there were 315 patients in the neurorehabilitation unit following different incidents, including vascular brain damage, brain injury, spinal cord injury, surgically treated patients with a brain tumor, sclerosis multiplex, myelopathy, and polyneuropathy. It was determined that 218 (65.71%) patients were stroke survivors, and the individuals were admitted to the neurorehabilitation ward immediately from the hospital, directly after the acute phase cessation, in a subacute phase of the stroke. A medical doctor assessed each patient at the beginning of the stay at the unit, and afterward, he/she was suggested to comprehensive individual and similar rehabilitation. Most of the patients were able to cooperate, understand instructions, and communicate. In addition, the majority of the individuals followed the same diet, including a baseline diet for most patients or a diet for people with diabetes mellitus. All the meals were prepared in the hospital and were distributed to the patients at the same time daily.

Decisively, 97 (30.79%) individuals with other diseases than stroke were excluded from the study, and 25 (11.47% of stroke survivors; 7.94% of all individuals rehabilitated at the health center fully completed the examination, saliva sampling, and were considered in the analysis (Table 1). The other stroke patients had to be excluded from the study because of the following reasons: 80 (36.70%) patients refused to participate in the study; 42 (19.27%) people were uncooperative since they could not give conscious written informed consent for participation in the research and/or entirely communicate; 28 (12.84%) patients did not report for a sampling of saliva and dental examination, although they gave informed consent and were reminded up to four times; 11 (5.04%) subjects withdrew from the research after sampling of non‐stimulated saliva due to physiological or psychological fatigue; 5 (2.29%) people were not able to gather the saliva due to issues concerning understanding the procedure because of cognitive and language deficits, and 2 (0.92%) patients were transferred to another hospital because of deterioration of general health.

**Table 1:** General characteristics of patients.

	Control *n* = 25		Stroke *n* = 25		p value
	Mean ± SD	95% CI	Mean ± SD	95% CI	
Sex (male/female)	12/13		12/13		> 0.9999
Age	63.52 ± 8.545	59.99 – 67.05	63.52 ± 8.545	59.99–67.05	> 0.9999
**Cognitive and physical functional status**					
ACE III	97.24 ± 1.422	96.65 – 97.83	69.8 ± 22.97	60.32–79.28	< 0.0001
BI	20 ± 0	20—20	10.92 ± 3.662	9.408–12.43	< 0.0001
BBS	55.52 ± 0.5099	55.31 – 55.73	31 ± 16.27	24.28–37.72	< 0.0001
FIM	125.3 ± 0.6904	125 – 125.6	86.88 ± 30.31	74.37–99.39	< 0.0001
**Comorbidities**					
Hypertension n (%)	18 (72)		17 (68)		> 0.9999
Diabetes n (%)	4 (16)		4 (16)		> 0.9999
Epilepsy n (%)	4 (16)		3 (12)		> 0.9999
Arteriosclerosis n (%)	7 (28)		8 (32)		> 0.9999
Limb thrombosis n (%)	2 (8)		2 (8)		> 0.9999
Atrial fibrillation n (%)	3 (12)		3 (12)		> 0.9999
**Drugs**					
< 5 drugs/day *n* (%)	10 (40)		9 (36)		> 0.9999
≥ 5 drugs/day *n* (%)	15 (60)		16 (64)		> 0.9999

*ACE III* Addenbrooke’s Cognitive Examination, *BI* Barthel Index, *BBS* Berg Balance Scale, *FIM* functional independence measure; differences statistically significant at: *p < 0.05; **p < 0.005; ***p < 0.0005; ****p < 0.0001.

Essential information on the general condition and health status of patients were obtained from individuals' files and contained: gender, age, the medication used, time since diagnosis of cerebral stroke, and medical history (Table 1).

For measurement of the functional status of the individuals the following scales were used.

1. Addenbrooke’s Cognitive Examination III (ACE III) for differentiation between patients without and with cognitive impairment^43^.
2. The Barthel Index (BI) to measure performance in activities of daily living (ADL)^44^.
3. The Berg Balance Scale (BBS) to determine an individual's ability or inability to safely balance during a series of predetermined tasks^45^.
4. The functional independence measure (FIM) for exploration of the patient's psychological, physical, and social functioning^46^.

One experienced medical doctor, a neurorehabilitation specialist, qualified all the patients for the examination according to study criteria (Table 2).

**Table 2:** Inclusion and exclusion criteria for the subjects in the study group.

Inclusion criteria	Exclusion criteria
Age of consent (> 18 years)	Poor general condition
Consciousness	Inadequate capacity to follow instructions, i.e., not understanding how to perform the procedures, inability to collect a saliva sample, and being unable to answer questions during the examination
Giving of written and informed consent for saliva sampling and oral examination	No recovery from the acute phase of ischemic or hemorrhagic stroke in all brain areas
Confirmed cerebral infarction or cerebral hemorrhage based on CT and magnetic resonance imaging (MRI)	Stroke recurrence during the subacute phase
Recovery from the acute phase of ischemic or hemorrhagic stroke in all brain areas	Insufficient cooperation due to cognitive and/or language deficits
The first admission to cure stroke unit was more than 5–6 (to 10) hours from the onset of the early neurological symptoms	Ischemic stroke treated with thrombectomy or thrombolysis
	Incapability to collect a saliva sample
	Legal guardianship
	Patients with psychiatric or cognitive disorders
	Autoimmune disease (rheumatoid arthritis, systemic lupus erythematosus)
	Lung disease (chronic obstructive pulmonary disease) or cardiovascular disease (angina or uncontrolled hypertension)
	Heart failure above NYHA II
	Cognitive impairment before stroke
	Intake of dietary supplements and vitamins for the last 3 months
	Smokers
	Patients with malnutrition, i.e., having body mass index < 18 kg/m^2^ or with weight loss > 10% during the previous 3 months

Clinical examination was performed and study material was collected in the subacute phase of stroke, between 30 and 35 days after the incident.

### Control group

The control group included generally healthy patients who reported for oral examination to the Department of Restorative Dentistry of the Medical University of Bialystok (Bialystok, Poland) from March to September 2020. From a group of 112 volunteers, 25 matched to the study group were selected after careful analysis of medical history and dental examinations. Controls were similar to the stroke patients regarding age and gender, oral hygiene, dentition, and periodontium status (Table 1). All participants were provided with medical clearance by clinicians before involvement in the research. Individuals from the control group followed a regular, balanced diet (which was not restricted), and they were given standard physical activity recommendations.

### Sampling of saliva

The research material was total mixed non‐stimulated saliva (NWS) and stimulated saliva (SWS), and both samples were collected via spitting. Saliva sampling was done in the health center during summertime, i.e., between June and September, to keep similar weather conditions outside. Before the procedure, individuals were instructed not to intake any solid food and/or liquid, other than clean water, at least two hours before saliva sampling. All individuals were in the subacute phase of the stroke. The study and control group individuals had not taken any medication eight hours before sampling of saliva^47,48^. The oral bioliquid was gathered, from subjects that had restrained from intensive physical activity for the preceding twelve hours, i.e., between 7:30 and 9:00 a.m. The patients were also indicated not to carry out any oral hygiene practices (i.e., mouth rinsing, teeth brushing, gum chewing, etc.)^48,49^. The saliva was collected after a 5‐min adaptation to the environment in a separate, private room. The oral cavity was rinsed two times with distilled water at room temperature before gathering saliva to avoid possible impurity from other sources. During the procedure, subjects were seated in an adjustable chair, individually adapted to each individual's height, with the head slightly bent downwards and resting in a convenient position, and they tried to limit their lips and face movements^48,49^. The samples of saliva that were stored during the first minute were ejected. Saliva was accumulated into a sterile Falcon tube placed in a container with ice. The non‐stimulated saliva was collected for 10 min and stimulated saliva was gathered in the same manner for 5 min to avoid the patients' physiological and/or psychological fatigue. The secretion of this oral bioliquid was stimulated by 10 μL of 2% citric acid applied to the tongue's central part every 30 s. Afterward, the volume of saliva was measured using a calibrated pipette with an accuracy of 0.1 mL^50^. The minute flow of NWS and SWS was measured by dividing the saliva volume by the time essential for its secretion (mL/min). Forthwith, after collection of saliva, it was centrifuged (+ 4 °C, 20 min, 3000×*g*; MPW 351, MPW Med. Instruments, Warsaw, Poland). To protect againts sample oxidation, butylated hydroxytoluene (BHT, Sigma‐Aldrich, Saint Louis, MO, USA) was added into the acquired supernatants in the amount of 10 μL 0.5 M BHT in acetonitrile (ACN)/1 mL of saliva^51^. The samples of NWS and SWS were frozen at − 80 °C and stored for no more than 3 months for further investigation.

### Oral examination

The dentition was evaluated in a separate room shortly after saliva sampling. According to the World Health Organization criteria, the individual's check-up was carried out using a plain mouth mirror and a dental probe in artificial lighting^52,53^. The dentist performed the dental examination in front of the chair where the patient was seated and with the individual's head resting against the wall. Each accessible tooth surface was assessed and scored as generally healthy, decayed (DT), extracted due to the carious process (MT), or filled because of caries (FT). The data collected were used to calculate the DMFT index, that expresses dental caries experience, and it is the sum of the following components: DT, MT, and FT. Additionally, the prevalence of dental caries was determined as a percentage of patients with DMFT > 0. Gingival index (GI) and Plaque Index (PlI) were evaluated on the teeth 16, 12, 24, 36, 32, 44 using four-degree scales (from 0 to 3)^54^. The oral examination was performed by two dentists (P.G. and K.G.), subsequently after sampling of non‐stimulated (NWS) and stimulated (SWS) whole saliva, and after previous training and calibration by an experienced dental specialist (A.Z.). The inter‐examiner and intra‐examiner agreement for DMFT, PlI and GI was evaluated by another dental examination in ten10 patients with a κ > 0.94.

### Patient’s consent and bioethical issues

All individuals, i.e., stroke patients and healthy controls, voluntarily took part in the study, and they were provided with comprehensive information concerning its purpose, procedures, risks, and benefits. Full written consent was obtained from all participants in accordance with the Declaration of Helsinki for a sampling of saliva and dental examination.

Before the research, it was approved by the Bioethics Committee of the Poznan University of Medical Sciences (resolutions 59/19 and 890/19).

### Salivary cytokine profile

Salivary concentrations of tumor necrosis factor α (TNF-α), interleukin 6 (IL-6), and interleukin 10 (IL-10) were assessed using commercial enzyme-linked immunosorbent assay (ELISA) kits according to the manufacturer’s instructions (EIAab, Wuhan, China). Briefly, specific antibodies suitably labeled with an enzyme were added to the antigen-coated plate. Depending on the amount of antigen, antibodies specifically bound to the antigen and unbound antibodies were eluted. After adding the substrate/chromophore, the enzyme-catalyzed the reaction, and the colored reaction product was measured colorimetrically at 405 nm using Mindray MR-96 Microplate Reader. All determinations were performed in duplicate samples.

Salivary total protein content was determined in duplicate samples by the bicinchoninic acid (BCA) method (Pierce BCA Protein Assay Kit; Rockford, IL, USA). All results were presented as concentration (pg/mL), salivary output (pg/min), and specific content (mg/mg protein). The TNF-α /IL-10 and IL-6/IL-10 ratios were also calculated.

### Statistical data analysis

Statistical analysis was carried out using GraphPad Prism 8 for macOS (GraphPad Software, La Jolla, CA, USA). Due to the fact that most of the results were characterized by a normal distribution (Kolmogorov–Smirnov test), the t-student test was used to compare two groups. If the distribution was not normal, the Mann–Whitney U test was used. ANOVA analysis of variance with Tukey's post hoc test was used to compare the three groups, and p values were calculated with correction for multiple comparisons. Correlations between salivary biomarkers and clinical data were performed using the Pearson correlation coefficient. The results were presented as mean ± SD and 95% confidence interval (95% CI). The assumed statistical significance was p < 0.05.

Cohen Kappa (online calculator) was used to establish the inter-and intracorporeal agreement between the evaluated dental indicators.

The diagnostic usefulness of salivary cytokines was determined using ROC (Receiver Operating Curve) analysis by evaluating the area under the curve (AUC). The sensitivity and specificity and the cut-off point, which was characterized by the highest sensitivity and specificity, were given.

The number of patients was set a priori based on our previous study. For this purpose, an online sample size calculator (ClinCalc) was used. The level of significance was set at 0.05, and the power of the study was 0.9. Variables used for sample size calculation were concentration of salivary IL-6 and IL-10. The minimum number of patients was 21 (for one group).

### Institutional Review Board statement

The study was conducted according to the guidelines of the Declaration of Helsinki, and approved by the Ethics Committee of the Poznan University of Medical Sciences (resolutions 59/19 and 890/19) in Poland.

### Informed consent

Informed consent was obtained from all subjects involved in the study.

## Results

### Salivary gland function

The secretion of non‐stimulated saliva did not differ significantly between the study and control groups. Stimulated saliva secretion was significantly decreased (↓36%) in the stroke population than the controls (Table 3).

**Table 3:** Salivary gland function and dental characteristics of the stroke and healthy controls.

	Control *n* = 25		Stroke *n* = 25		p value
	Mean ± SD	95% CI	Mean ± SD	95% CI	
**NWS**					
Flow rate (mL/min)	0.33 ± 0.08461	0.2951–0.3649	0.394 ± 0.2459	0.2925–0.4955	0.2245
Total protein (μg/mL)	1220 ± 174	1148–1292	1141 ± 281.5	1025–1257	0.2391
**SWS**					
Flow rate (mL/min)	0.9112 ± 0.2629	0.8027–1.02	0.5796 ± 0.2454	0.4783–0.6809	< 0.0001
Total protein (μg/mL)	1310 ± 172.3	1239–1382	947 ± 244.8	846–1048	< 0.0001
DMFT	24.4 ± 7.848	21.16–27.64	23.08 ± 7.989	19.78–26.38	0.5584
GI	0.8136 ± 0.8085	0.4799–1.147	0.72 ± 0.7294	0.4189–1.021	0.6693
PlI	1.254 ± 0.9743	0.8514–1.656	1.234 ± 0.9953	0.8228–1.644	0.9431

*NWS* non-stimulated whole saliva; *SWS* stimulated whole saliva; *DMFT index* a sum of decayed teeth (DT), teeth missing due to carious process (MT), and teeth filled because of caries (FT); *GI* Gingival Index; *PlI* Plaque Index.

The concentration of total protein in non‐stimulated saliva did not differ significantly between healthy controls and stroke individuals. The total protein concentration in stimulated saliva of the stroke population was significantly lower (↓28%) than in the control group (Table 3).

### Oral health status

DMFT, GI, and PlI were not significantly different between the study and control group (Table 3).

### Salivary cytokines

Non-stimulated saliva of stroke patients was characterized by significantly higher concentration (↑135%), output (↑196%), and specific amount (↑170%) of TNF-α. Similarly, these parameters were increased regarding IL-6 (respectively: ↑85%, ↑124%, 103%). On the other hand, the concentration (↓47%), output (↓78%), and specific amount (↓42%) of IL-10 decreased. As a result of these changes, the TNF-α/IL-10 ratio (↑507%) and IL-6/IL-10 ratio (↑378%) increased markedly.

The results of measurements in stimulated saliva were comparable to those in non-stimulated saliva. The concentration (↑68%) and specific amount (↑147%) of TNF-α were significantly higher in patients with stroke than in control. Mentioned parameters were also higher in relation to IL-6 (concentration: ↑62%, specific amount: ↑57%). The output of TNF-α and IL-6 remained statistically insignificant. IL-10 concentration, output, and specific amount decreased, similarly to NWS (respectively: ↓65%, ↑36%, ↑48%). In consequence, TNF-α/IL-10 (↑611%) and IL-6/IL-10 (↑568%) ratios increased greatly (Table 4).

**Table 4:** Salivary cytokines in stroke patients and healthy controls.

Salivary biomarker	NWS					SWS				
	Control *n* = 25		Stroke *n* = 25		p value	Control *n* = 25		Stroke *n* = 25		p value
	Mean ± SD	95% CI	Mean ± SD	95% CI		Mean ± SD	95% CI	Mean ± SD	95% CI	
**TNF-α**										
Concentration (pg/mL)	11.72 ± 3.503	10.27–13.16	27.93 ± 6.021	25.44–30.41	< 0.0001	4.02 ± 0.6272	3.761–4.279	6.776 ± 0.8391	6.43–7.122	< 0.0001
Output (pg/min)	3.731 ± 1.181	3.244–4.218	11.07 ± 7.472	7.989–14.16	< 0.0001	3.649 ± 1.189	3.158–4.14	3.947 ± 1.768	3.217–4.677	0.4884
Specific amount (ng/mg protein)	9.846 ± 3.591	8.364–11.33	26.63 ± 11.21	22–31.25	< 0.0001	3.106 ± 0.5689	2.871–3.341	7.702 ± 2.53	6.658–8.746	< 0.0001
**IL-6**										
Concentration (pg/mL)	15.81 ± 3.715	14.28–17.34	29.27 ± 9.684	25.28–33.27	< 0.0001	6.996 ± 0.8605	6.64–7.351	11.3 ± 1.065	10.86–11.74	< 0.0001
Output (pg/min)	5.213 ± 1.863	4.443–5.982	11.68 ± 8.251	8.27–15.08	0.0004	6.37 ± 2.004	5.543–7.197	6.487 ± 2.655	5.391–7.583	0.8614
Specific amount (ng/mg protein)	13.27 ± 3.674	11.75–14.79	26.89 ± 9.536	22.95–30.82	< 0.0001	5.449 ± 1.094	4.997–5.9	12.8 ± 3.838	11.21–14.38	< 0.0001
**IL-10**										
Concentration (pg/mL)	3.934 ± 0.7284	3.634–4.235	2.078 ± 0.8456	1.728–2.427	< 0.000	0.5012 ± 0.07068	0.4721–0.5304	0.1753 ± 0.085	0.1402–0.2104	< 0.0001
Output (pg/min)	0.4502 ± 0.1308	0.3962–0.5042	0.09977 ± 0.05173	0.07842–0.1211	0.0031	1.287 ± 0.3666	1.136–1.438	0.8238 ± 0.6476	0.5564–1.091	< 0.0001
Specific amount (ng/mg protein)	3.303 ± 0.8475	2.953–3.653	1.927 ± 0.9295	1.543–2.31	< 0.0001	0.3881 ± 0.07196	0.3584–0.4178	0.2015 ± 0.1105	0.1559–0.2471	< 0.0001
**TNF-α/IL-10**										
Ratio	3.119 ± 1.285	2.588–3.649	18.96 ± 17.54	11.71–26.2	< 0.0001	8.186 ± 1.776	7.452–8.919	58.24 ± 56.44	34.94–81.54	< 0.0001
**IL-6/IL-10**										
Ratio	4.156 ± 1.28	3.627–4.684	19.88 ± 22.59	10.56–29.21	0.0011	14.33 ± 3.247	12.99–15.67	95.8 ± 86.71	60.01–131.6	< 0.0001

*TNF-α* tumor necrosis factor α, *IL-6* interleukin 6, *IL-10* interleukin 10.

### Correlations

Statistically significant correlations were summed up in Table 5. The positive correlations were revealed only in non-stimulated saliva between IL-6 concentration and ADL, and IL-6 specific amount and ADL.

**Table 5:** Statistically significant correlations in patients with ischemic stroke.

Pair of variables	r	p value
**NWS**		
TNF-α concentration and BBS	− 0.612	0.001
TNF-α concentration and BI	− 0.789	< 0.001
TNF-α concentration and ACE III	− 0.832	< 0.001
TNF-α output and BBS	− 0.398	0.045
IL-6 concentration and BI	0.464	0.02
IL-6 output and FIM	− 0.41	0.042
IL-6 specific amount and PlI	− 0.481	0.015
IL-6 specific amount and BI	0.418	0.037
**SWS**		
TNF-α output and BBS	− 0.474	0.017
TNF-α output and BI	− 0.761	< 0.001
TNF-α output and ACE III	− 0.801	< 0.001
IL-6 output and BBS	− 0.512	0.009
IL-6 output and BI	− 0.796	< 0.001
IL-6 output and ACE III	− 0.754	< 0.001

*NWS* non-stimulated whole saliva, *SWS* stimulated whole saliva, *TNF-α* tumor necrosis factor α, *IL-6* interleukin 6, *PLI* Plaque Index, *ACE III* Addenbrooke’s Cognitive Examination, *FIM* functional independence measure, *BBS* Berg Balance Scale.

The strong negative correlations in non-stimulated saliva were observed between TNF-α concentration and BBS, TNF-α concentration and ACE III or TNF-α concentration and ADL. Moreover, IL-6 specific amount and PlI and IL-6 output and FIM correlate negatively in unstimulated saliva. The negative correlation between TNF-α output and BBS was noted in either stimulated or non-stimulated saliva.

In stimulated saliva, TNF-α output correlated negatively with BBS, ADL and ACE III. On the other hand, IL-6 output was observed to correlate negatively with BBS and ADL.

Importantly, we did not show any relationship between salivary cytokine levels and time of stroke diagnosis.

In the control group, we did not observe any statistically significant correlations between cytokine content and clinical parameters.

### ROC analysis

The results of ROC analysis were presented in Tables 6 and 7.

**Table 6:** Receiver operating characteristics of salivary cytokines in non-stimulated saliva of stroke patients and healthy controls.

Salivary biomarker	NWS							
	AUC	95% CI	p-value	Cut off	Sensitivity%	95% CI	Specificity%	95% CI
**TNF-α**								
Concentration (pg/mL)	0.992	0.9760–1.000	< 0.0001	> 17.46	96	80.46–99.79	96	80.46–99.79
Output (pg/min)	0.8544	0.7470–0.9618	< 0.0001	> 4.727	76	56.57–88.50	80	60.87–91.14
Specific amount (ng/mg protein)	0.9712	0.9340–1.000	< 0.0001	> 15.61	92	75.03–98.58	92	75.03–98.58
**IL-6**								
Concentration (pg/mL)	0.8976	0.7944–1.000	< 0.0001	> 22.08	84	65.35–93.60	96	80.46–99.79
Output (pg/min)	0.7376	0.5897–0.8855	0.004	> 5.602	76	56.57–88.50	72	52.42–85.72
specific amount (ng/mg protein)	0.9104	0.8168–1.000	< 0.0001	> 18.03	84	65.35–93.60	92	75.03–98.58
**IL-10**								
Concentration (pg/mL)	0.9568	0.9078–1.000	< 0.0001	< 3.014	88	70.04–95.83	92	75.03–98.58
Output (pg/min)	0.776	0.6400–0.9120	0.0008	< 1.140	80	60.87–91.14	68	48.41–82.79
specific amount (ng/mg protein)	0.8848	0.7883–0.9813	< 0.0001	< 2.173	72	52.42–85.72	96	80.46–99.79
TNF-α/IL-10	0.9984	0.9931–1.000	< 0.0001	> 5.577	100	86.68–100.0	96	80.46–99.79
IL-6/IL-10	0.9792	0.9426––1.000	< 0.0001	> 7.625	92	75.03–98.58	100	86.68–100.0

TNF-α–tumor necrosis factor α; IL-6 – interleukin 6; IL-10 – interleukin 10.

**Table 7:** Receiver operating characteristics of salivary cytokines in stimulated saliva of stroke patients and healthy controls.

Salivary biomarker	SWS							
	AUC	95% CI	p-value	Cut off	Sensitivity%	95% CI	Specificity%	95% CI
**TNF-α**								
Concentration (pg/mL)	0.9984	0.9931–1.000	< 0.0001	> 4.941	100	86.68–100.0	96	80.46–99.79
Output (pg/min)	0.5616	0.3950–0.7282	0.4551	> 4.211	52	33.50–69.97	76	56.57–88.50
Specific amount (ng/mg protein)	1	1.000–1.000	< 0.0001	> 4.249	100	86.68–100.0	100	86.68 -100.0
**IL-6**								
Concentration (pg/mL)	1	1.000–1.000	< 0.0001	> 8.986	100	86.68–100.0	100	86.68–100.0
Output (pg/min)	0.536	0.3704–0.7016	0.6624	> 6.640	60	40.74–76.60	60	40.74–76.60
Specific amount (ng/mg protein)	0.9968	0.9882–1.000	< 0.0001	> 8.366	96	80.46–99.79	100	86.68–100.0
**IL-10**								
Concentration (pg/mL)	0.9984	0.9931–1.000	< 0.0001	< 0.3165	96	80.46–99.79	100	86.68–100.0
Output (pg/min)	1	1.000–1.000	< 0.0001	< 0.2250	100	86.68–100.0	100	86.68–100.0
Specific amount (ng/mg protein)	0.9136	0.8353–0.9919	< 0.0001	< 0.2665	76	56.57–88.50	96	80.46–99.79
TNF-α/IL-10	1	1.000–1.000	< 0.0001	> 17.01	100	86.68–100.0	100	86.68–100.0
IL-6/IL-10	1	1.000–1.000	< 0.0001	> 24.60	100	86.68–100.0	100	86.68–100.0

*TNF-α* tumor necrosis factor α, *IL-6* interleukin 6, *IL-10* interleukin 10.

The majority of analyzed cytokines significantly differentiated the patients with ischemic stroke from healthy controls. What is worth mentioning, many of given parameters in stimulated saliva, were characterized by AUC = 1, sensitivity and specificity reaching up to 100%, e.g. TNF-α/IL-10 ratio, IL-6/IL-10 ratio, TNF-α specific amount, and IL-6 concentration. In non-stimulated saliva, TNF-α/IL-10 and IL-6/IL-10 ratios distinguished themselves with high AUC and both great sensitivity and specificity.

### TNF-α diagnostic significance

**Figure 1:**
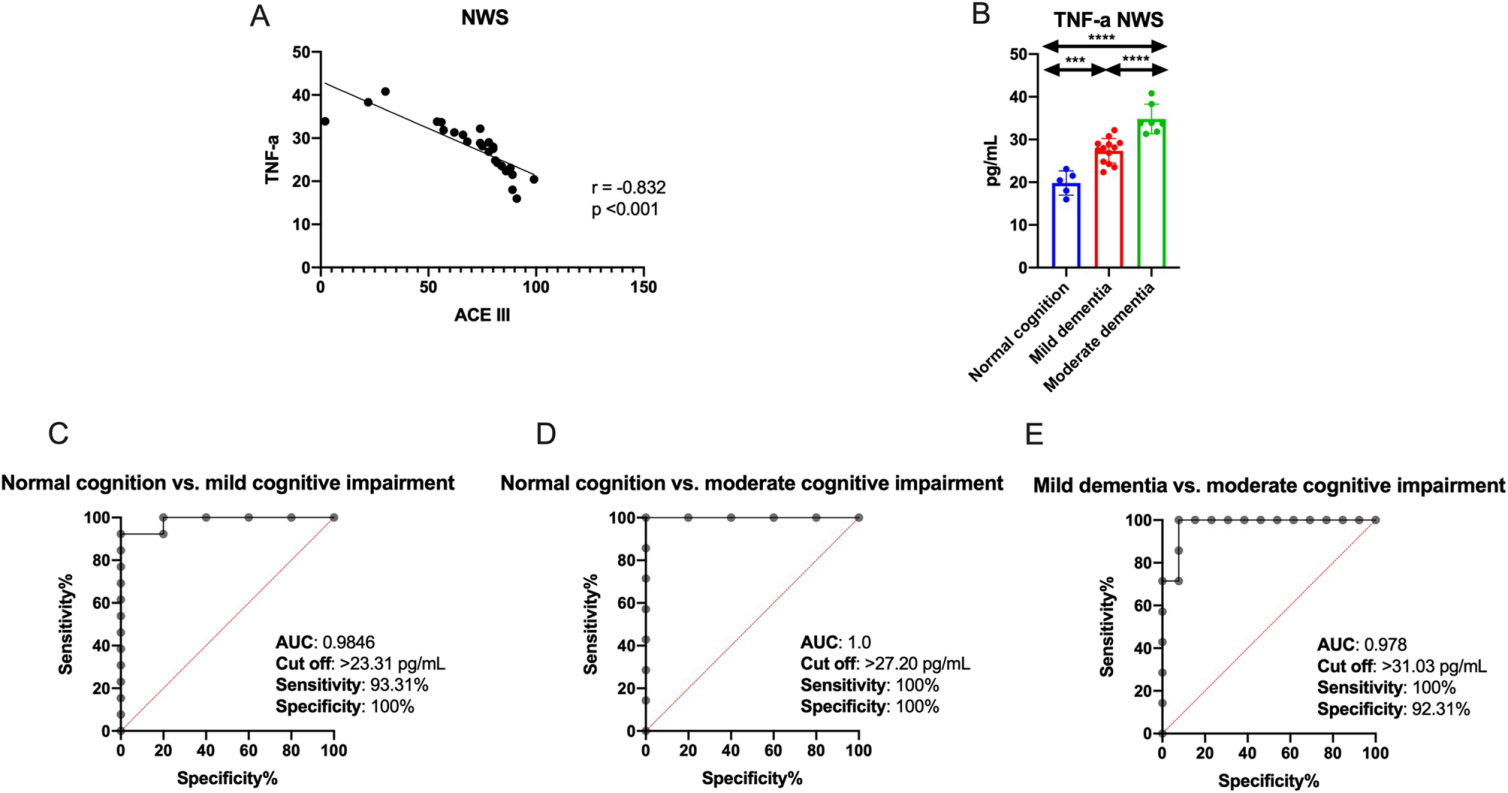
Diagnostic significance of salivary TNF-α in ischemic stroke patients: correlation between salivary TNF-α and cognitive function in ACE III scale (**A**), results of ROC analysis for salivary TNF-α in relation to cognitive function status in ACE III scale (**B**–**E**). Differences statistically significant at: **p < 0.005, ****p < 0.0001.

Salivary TNF correlates remarkably negatively with cognitive function in the ACE III scale. Therefore, we examined how this parameter differentiates stroke patients according to cognitive impairment. For this purpose, stroke patients were divided into three groups: normal cognitive function (100–89 in the ACE III scale), mild cognitive impairment (88–61 in the ACE III scale), and moderate cognitive impairment (< 61 in the ACE III scale)^55,56^. Salivary TNF-α differentiates (with high sensitivity and specificity) stroke patients with normal cognitive function from patients with mild to moderate cognitive impairment (Fig. 1).

## Discussion

Our study is the first to evaluate the salivary cytokine profile in patients with ischemic stroke. We have demonstrated that assessment of TNF-α, IL-6, and IL-10 levels in both NWS and SWS significantly differentiates stroke patients from healthy controls. However, particular attention should be paid to salivary TNF-α, which may be a non-invasive biomarker of cognitive function/physical status in stroke subjects.

In recent years, increasing attention has been given to the potential use of saliva in laboratory diagnostics. This biomaterial allows for repeated, non-invasive, and stress-free sampling, which is especially important in disabled and elderly patients^57^. Saliva is also a non-infectious and relatively durable bioliquid^32,58^. The concentration of many biomarkers in saliva highly correlates with their blood/CSF levels, allowing saliva to be used in the diagnosis of various systemic diseases^59–62^. Indeed, saliva can be an alternative to blood for the determination of enzymes, hormones, immunoglobulins, and redox biomarkers^63,64^. Saliva has also been shown to be valuable in assessing cytokines, chemokines, pro-inflammatory enzymes, and growth factors^65–67^. Although salivary cytokines' diagnostic utility has been demonstrated in metabolic and neurodegenerative diseases^38–42^, there are no data on ischemic stroke patients.

We showed that salivary TNF-α and IL-6 were significantly higher, whereas IL-10 content was statistically lower in ischemic stroke patients than healthy controls. TNF-α/IL-10 and IL-6/IL-10 ratio were also significantly higher in NWS and SWS of stroke patients, indicating an intensification of the inflammatory process and a limited anti-inflammatory role of IL-10. As we have shown by ROC analysis, salivary biomarkers are also characterized by a high diagnostic utility in stroke diagnostics. Salivary TNF-α, IL-6, and IL-10 as well as TNF-α/IL-10 and IL-6/IL-10 ratio significantly distinguish stroke patients from controls with high sensitivity and specificity. This fact should not be surprising because inflammatory and immune factors have been indicated as potential stroke biomarkers^68,69^. Indeed, in stroke patients, activation of CNS cells (mainly microglia and astrocytes) and increased expression of blood cytokines, interleukins, chemokines, adhesion molecules, and pro-inflammatory enzymes is observed^70^. Interestingly, the severity of inflammation correlates with brain infarct size and neurological deficits in CT and physical examination^71–73^. However, cytokines' diagnostic utility has only been demonstrated in plasma/serum and CSF of ischemic stroke patients^73–75^. Our study is the first to show the utility of saliva to determine cytokine levels in stroke subjects. This raises hopes for non-invasive, painless, and non-stressful stroke diagnostics. It is believed that cytokines synthesized in the stroke focus pass through the damaged BBB into the blood and then into saliva by passive transport (diffusion or ultrafiltration) or by a specific transporter (facilitated diffusion or active transport)^76,77^. The BBB has a prominent role in the development of the inflammatory response by increasing the cytokine circulation between the CSF and the bloodstream^9^. Unfortunately, in our research, we did not assess the cytokine content in blood, so further studies are needed to evaluate stroke patients' saliva-blood relationship. Nevertheless, it can be speculated that, as in other CNS diseases, salivary cytokine concentrations reflect their plasma/CSF levels^28,31,78^.

In our patients, the content of pro-inflammatory TNF-α and IL-6 was higher, and anti-inflammatory IL-10 was lower in both NWS and SWS of stroke patients. However, it is important to note that saliva's cytokine profile depends on the type of salivary gland involved in saliva secretion. Indeed, up to 70% of non-stimulated saliva is produced in the submandibular glands, making the NWS composition very similar to blood plasma. During stimulation, the parotid gland's contribution to saliva formation increases^79^. Stimulated saliva is characterized by significantly higher concentrations of Na^+^, HCO_3_^−^ and Cl^−^ ions and lower contents of K^+^ and phosphate ions and mucins compared to non-stimulated saliva^80^. Cytokine production in salivary gland cells also increases, making the composition of SWS more different from that of plasma^81^. Therefore, evaluation of cytokines in NWS rather than in SWS may be of greater diagnostic value. In our study, among all assessed cytokines, tumor necrosis factor α deserves special attention. Salivary TNF-α differentiates stroke patients from controls with high sensitivity and specificity and correlates with cognitive dysfunction in the ACE III scale and severity of functional impairment in the BBS and ADL scale. Therefore, in the next step, we examined how this parameter distinguishes stroke patients according to cognitive dysfunction. For this purpose, stroke subjects were divided into three groups: normal cognition (100–89 in the ACE III scale), mild cognitive impairment (88–61 in the ACE III scale), and moderate cognitive impairment (< 61 in the ACE III scale)^55,56^. Salivary TNF-α differentiates (with high sensitivity and specificity) stroke patients with normal cognitive function from patients with mild to moderate cognitive decline (Fig. 1). Although further studies in a larger patient population are needed, TNF-α may be a potential biomarker of cognitive impairment/functional status in stroke cases. As shown in previous studies, elevated TNF-α serum level was noted already in the first few hours after the ischemic stroke^10,16^. Serum TNF-α also correlates with brain infarct volume and severity of neurological symptoms as well as reflects the degree of patients' functional disability^82,83^. Indeed, TNF-α has a unique role in stroke pathogenesis. Primarily, it is the first inflammatory mediator produced in the ischemic area^6,83^. Secondly, it stimulates the synthesis/secretion of chemotactic factors that stimulate peripheral blood leukocytes and activates other cytokines like IL-1, IL-6, and interferon-gamma (IFN-γ)^10^. TNF-α also enhances MMPs activity^84^, thereby increasing endothelial cell permeability and BBB dysfunction^85^. Our study is the first to demonstrate that salivary TNF-α can be a potential biomarker of stroke patient’s functioning. Interestingly, the level of TNF-α in NWS correlates more strongly with the cognitive and physical functional status of stroke patients. Thus, non-stimulated saliva has a better diagnostic potential than SWS. However, further studies are needed to evaluate the relationship between stroke location, brain infarct volume, severity of neurological symptoms and salivary cytokine levels.

Despite the unquestionable advantages, the use of saliva in diagnostics also has some drawbacks. The biomarker concentration in saliva may be highly dependent on the degree of salivary gland secretion^86^. Because salivary hypofunction may occur in stroke patients^87,88^, in our study, the concentration of inflammatory biomarkers was standardized to total protein content and salivary flow rate. Nevertheless, both the concentration (pg/mL), salivary output (pg/min), and specific content (per mg protein) of TNF-α and IL-6 were significantly higher, whereas IL-10 was statistically lower in NWS and SWS of stroke patients compared to controls. Importantly, we did not observe a relationship between salivary gland function and the assessed cytokines. This suggests a negligible effect of salivary hypofunction on salivary TNF-α, IL-6, and IL-10 levels. Nevertheless, aging, medications, diet supplements, smoking, as well as oral and systemic diseases can also modify the cytokine profile of saliva^89–91^. At this time, it is important to emphasize the careful selection of the control group in the presented study. Control individuals are matched not only by age and sex to the study group but also by other clinical features, including concomitant diseases, medications, oral hygiene status, etc. This significantly reduces the impact of confounding factors on the obtained results.

Unfortunately, our study has a number of limitations. Although the main source of cytokines in saliva is their diffusion from plasma^92^, we did not evaluate cytokine content in blood. Further studies are needed to evaluate saliva-blood correlations of inflammatory mediators, as well as to clarify the clinical utility of these biomarkers according to stroke subtype and location. Studies on larger numbers of patients are also necessary. However, our study is the first to indicate the clinical potential of salivary cytokines in the diagnosis of stroke patients.

In summary, stroke patients have increased salivary secretion of pro-inflammatory cytokines and decreased anti-inflammatory mediators. Salivary TNF-α, IL-6, and IL-10 may be potential non-invasive biomarkers that distinguish stroke patients from controls with high sensitivity and specificity. Of particular note is salivary TNF-α, which may indicate cognitive/physical impairment in post-stroke individuals. Thus, salivary cytokines could support diagnostic imaging in stroke patients. Further studies are needed in a larger stroke population to assess cytokines at both the salivary and central (plasma/serum) levels. Evaluation of other inflammatory mediators for their potential diagnostic significance also requires research.

## Data Availability

The datasets generated for this study are available on request to the corresponding author.
